# *In vitro* differences in toddalolactone metabolism in various species and its effect on cytochrome P450 expression

**DOI:** 10.1080/13880209.2022.2108062

**Published:** 2022-08-09

**Authors:** Lina Shan, Xianbao Shi, Tingting Hu, Jiayin Hu, Zhe Guo, Yonggui Song, Dan Su, Xiaoyong Zhang

**Affiliations:** a The First Affiliated Hospital of Jinzhou Medical University, Jinzhou, China; b Jiangxi University of Traditional Chinese Medicine, Nanchang, China

**Keywords:** Liver microsomes, species difference, enzyme kinetics, pharmacokinetics, LC-MS/MS

## Abstract

**Context:**

Toddalolactone, the main component of *Toddalia asiatica* (L.) Lam. (Rutaceae), has anticancer, antihypertension, anti-inflammatory, and antifungal activities.

**Objective:**

This study investigated the metabolic characteristics of toddalolactone.

**Materials and methods:**

Toddalolactone metabolic stabilities were investigated by incubating toddalolactone (20 μM) with liver microsomes from humans, rabbits, mice, rats, dogs, minipigs, and monkeys for 0, 30, 60, and 90 min. The CYP isoforms involved in toddalolactone metabolism were characterized based on chemical inhibition studies and screening assays. The effects of toddalolactone (0, 10, and 50 µM) on CYP1A1 and CYP3A5 protein expression were investigated by immunoblotting. After injecting toddalolactone (10 mg/kg), *in vivo* pharmacokinetic profiles using six Sprague–Dawley rats were investigated by taking 9-time points, including 0, 0.25, 0.5, 0.75, 1, 2, 4, 6 and 8 h.

**Results:**

Monkeys showed the greatest metabolic capacity in CYP-mediated and UGT-mediated reaction systems with short half-lives (*T*_1/2_) of 245 and 66 min, respectively, while *T*_1/2_ of humans in two reaction systems were 673 and 83 min, respectively. CYP1A1 and CYP3A5 were the major CYP isoforms involved in toddalolactone biotransformation. Induction of CYP1A1 protein expression by 50 μM toddalolactone was approximately 50% greater than that of the control (0 μM). Peak plasma concentration (*C*_max_) for toddalolactone was 0.42 μg/mL, and *T*_max_ occurred at 0.25 h post-dosing. The elimination *t*_1/2_ was 1.05 h, and the AUC_0–t_ was 0.46 μg/mL/h.

**Conclusions:**

These findings demonstrated the significant species differences of toddalolactone metabolic profiles, which will promote appropriate species selection in further toddalolactone studies.

## Introduction

**Figure 1.**
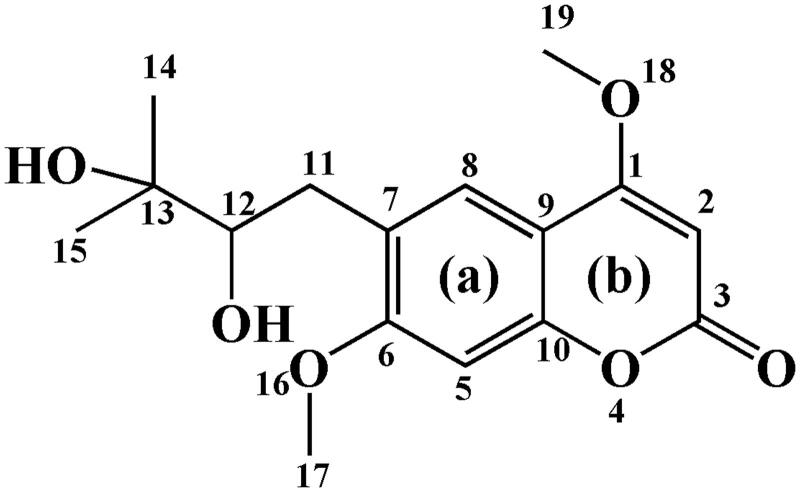
Structure of toddalolactone.

*Toddalia asiatica* (L.) Lam. (Rutaceae), is a woody climber widely distributed in southern China. Its roots and stem have been used as a folk medicine for dispelling pathogenic wind and pain, eliminating stasis and hemostasis, subduing swelling, and detoxicating (Zhang et al. 2017). Modern pharmacologic research has confirmed that *T. asiatica* extracts have multiple biological activities, including antitumor (Li et al. 2018; Zhou et al. 2019), anti-inflammatory (Kumagai et al. 2018), antiviral (Tang et al. 2016), and antibacterial (Murugan et al. 2015; He et al. 2018) effects. Toddalolactone (Figure 1) is the main component of *T. asiatica* (Ishii et al. 1991). Its pharmacological activities have been reported, including anticancer, antihypertension, anti-inflammatory, and antifungal activities (Zhang et al. 2014; Ni et al. 2020). In addition, toddalolactone exhibits significant spasmolytic activity which can be used to treat gynecological diseases (Lakshmi et al. 2002). Toddalolactone as a PAI-1 inhibitor might promote blood circulation and remove stasis (Yu et al. 2017).

Animal models are important tools for predicting the kinetics and toxicity of various medicines in humans. However, the content and the activity of metabolic enzymes in the liver vary greatly among different species (Shimada et al. 1997). Many studies have shown species-related differences in response to drug effects and toxicities in experimental animals and humans (Matal et al. 2008; Oesch et al. 2018; Straniero et al. 2020). Thus, comparing metabolic characteristics among different species can help to identify animal species with metabolic characteristics similar to humans that may be suitable for future *in vivo* studies (Zielinski and Mevissen 2015).

Cytochrome P450 (CYP) is an important phase I enzyme responsible for the metabolism of more than 70% of drugs and xenobiotics (Shi et al. 2016; Tang et al. 2017). The species-specific isoforms of CYP1A, CYP2C, CYP2D and CYP3A show appreciable interspecies differences in terms of catalytic activity, giving rise to species-dependent variations in drug metabolism and clearance (Martignoni et al. 2006). Therefore, identifying enzyme(s) involved in the biotransformation of toddalolactone can help predict interspecies differences in toddalolactone metabolism and avoid adverse drug reactions and herb-drug interactions.

The present study was performed to compare the *in vitro* metabolic characteristics of toddalolactone among seven species. In addition, liver microsomes and recombinant human supersomes were used to confirm metabolic enzymes involved in the biotransformation of toddalolactone.

## Materials and methods

### Chemicals and reagents

Toddalolactone (purity >98%) was purchased from Chengdu Pulisi Biotechnology Co., Ltd. (Chengdu, China). Furafylline, sulphaphenazole, quinidine, clomethiazole and omeprazole were obtained from CN Biomedicals, Inc. (Aurora, OH). Clobetasol propionate was purchased from J&K Scientific (Beijing, China). NADP^+^, D-glucose-6-phosphate (G-6-P), glucose-6-phosphate dehydrogenase (G-6-PD), uridine 5′-diphosphoglucuronic acid (UDPGA) (trisodium salt) and Tris-HCl were purchased from Sigma-Aldrich (St. Louis, MO). Acetonitrile (HPLC grade) and formic acid were purchased from Merck (Darmstadt, Germany). Other reagents and chemicals were of the highest quality available.

### Liver microsomes and recombinant human CYP

Thirteen recombinant human CYP (rhCYP) isoforms, including CYP1A1, CYP1B1, CYP1A2, CYP3A4, CYP3A5, CYP2C8, CYP2B6, CYP2C9, CYP2D6, CYP2A6, CYP2C19, CYP2E1 and CYP4F2, were purchased from Corning Life Sciences (Corning, MA). Mixed liver microsomes from beagle dogs (DLMs), rats (RLMs), mini pigs (PLMs), monkeys (MLMs), mice (MIMs), rabbits (RAMs) and humans (HLMs) were obtained from the Research Institute for Liver Disease Co., Ltd. (Shanghai, China). All the liver microsomes and rhCYP were stored at −80 °C until use.

### Incubation conditions in phase I (CYP) and II (UGT) reaction systems

The CYP reaction system (200 μL) consisted of 0.1 M potassium phosphate buffer (pH 7.4), an NADPH-generating system (1 mM NADPH, 10 mM G-6-P, 1 unit/mL G-6-PD, 4 mM MgCl_2_), and liver microsomes or recombinant human microsomes (supersomes). Each of the *in vitro* enzymatic assays using liver microsomes was linear with respect to incubation time and amount of protein. After a pre-incubation at 37 °C for 3 min, the reactions were initiated by adding NADP^+^. After incubating for 60 min, the reactions were quenched by adding 200 μL of ice-cold acetonitrile. Samples were centrifuged at 18,000 *g* for 15 min at 4 °C and 10 μL of supernatant was analyzed using an HPLC system. NADPH or CYP enzyme sources were not used in the control group to ensure that the metabolic reactions were CYP- and NADPH-dependent. All incubation assays were completed in triplicate. The data are presented as mean ± standard deviation (SD).

In the UGT reaction system, liver microsomes were incubated in 200 μL of the reaction system containing 4 mM UDPGA, 5 mM MgCl_2_, 20 µg/mL alamethicin, and 50 mM Tris-HCl buffer (pH 7.4). After pre-incubation at 37 °C for 3 min, UDPGA was added to the mixture to initiate the reactions. The mixtures were incubated at 37 °C for 60 min, followed by the addition of 200 μL of cold methanol to terminate the reactions. After centrifugation at 18,000 *g* for 15 min, 10 μL of the supernatant was analyzed by HPLC.

### Metabolic characteristics of toddalolactone in different liver microsomes

Toddalolactone (20 μM) was incubated with different liver microsomes including HLMs, PLMs, MLMs, RAMs, DLMs, RLMs and MIMs. The incubation reactions included oxidative and glucuronide conjugative metabolism. The incubation conditions are described above. Metabolite peaks were monitored, and the metabolic characteristics of toddalolactone in different liver microsomes were evaluated.

### Metabolic stability assessment with different microsomes

Toddalolactone (20 μM) was incubated with different liver microsomes, including HLMs, DLMs, PLMs, MLMs, RAMs, RLMs or MIMs in oxidative metabolism and glucuronidation reaction systems. The incubation volume was 600 μL, and the incubation conditions were as described above. The reaction was started by adding NADPH or UDPGA after pre-incubation at 37 °C for 3 min. Aliquots (100 μL) were removed after 0, 30, 60 or 90 min and mixed with 200 μL of acetonitrile. The mixtures were centrifuged at 18,000 *g* for 15 min at 4 °C and then 10 μL of the supernatant was analyzed by HPLC. The *in vitro T*_1/2_ (min) was calculated based on the slope of the linear regression of the natural logarithm of the parent remaining percentage versus incubation time using the formula *T*_1/2_ = ln2/slope (Huang and Ho 2009). All of the incubations were carried out in three independent experiments in duplicate.

### Kinetic studies

The enzyme kinetics of toddalolactone in oxidative metabolism and glucuronidation were determined by incubating liver microsomes (0.2 mg/mL) or CYPs (15 nM) with different concentrations of toddalolactone (1–200 μM). The kinetic parameters including *K*_m_ and *V*_max_ were obtained by fitting the experimental data to the Michaelis–Menten equation (Equation (1)), biphasic kinetics equation (Equation (2)), or substrate inhibition equation (Equation (3)) (He et al. 2018), and the results were graphically represented on Eadie–Hofstee plots. The *in vitro* intrinsic clearance (CL_int_) was calculated based on the *V*_max_/*K*_m_ ratio. Metabolites were quantified using the standard curve for toddalolactone because of the absence of metabolite standards. All incubations were repeated in three independent experiments. Kinetic constants were expressed with mean ± SD:

(1) $$V=\frac{V_{\max}\times [\text{S}]}{K_{m}+[\text{S}]}$$

(2) $$V=\frac{V_{\max1}\times [\text{S}]}{K_{s1}+[\text{S}]}+\frac{V_{\max2}\times [\text{S}]}{K_{s2}+[\text{S}]}$$

(3) $$V=\frac{V_{\max}\times [\text{S}]}{K_{s}+[\text{S}]+{\left[\text{S}\right]}^{2}/Ksi}$$

where *v* is the rate of reaction, *V*_max_ is the estimated maximum velocity, [S] is the substrate concentration, and *K*_m_ is the affinity constant of the substrate; *V*_max1_ and *V*_max2_ represent the estimated maximum velocity of the two metabolic phases; *K*_s1_ and *K*_s2_ represent the affinity constants for two metabolic phases; *K*_s_ and *K*si are the substrate affinity and inhibition constants, respectively.

### Prediction of *in vivo* hepatic clearance

The *in vivo* hepatic clearances in oxidative metabolism and glucuronidation of toddalolactone were calculated using Equations (4)–(6) (Li et al. 2008):

(4) $${\text{CL}}_{\mathrm{int}\;in\;vitro}=\sum_{i=1}^{\text{n}}\frac{V_{\max(Mi)}}{K_{m(Mi)}}$$

(5) $${\text{CL}}_{\mathrm{int}\;in\;vivo}={\text{CL}}_{\mathrm{int}\;in\;vitro}\cdot \text{SF}$$

(6) $${\text{CL}}_{H}=\frac{{\text{Q}}_{H}\cdot f_{u}\cdot {\text{CL}}_{\mathrm{int}\;in\;vivo}}{{\text{Q}}_{H}+f_{u}\cdot {\text{CL}}_{\mathrm{int}\;in\;vivo}}$$

where SF is the milligrams of microsomal protein per gram of liver multiplied by the grams of liver weight; CL*_H_* is hepatic clearance; *fu* is the unbound fraction in the blood (the value of *fu* was set to 1 due to the absence of available data for toddalolactone); *Q_H_* is the blood flow in the liver; and *Mi* is the metabolite of toddalolacone. The physiological parameters to calculate intrinsic clearance were as follows. The liver weight of dogs, rats and humans were 32, 40 and 25.7 g/kg body weight, respectively; the content of microsomal protein was 77.9, 44.8 and 48.8 mg/g of liver; and the blood flow in the liver was 30.9, 55.2 and 20.7 mL/(min ⋅ kg), respectively (Naritomi et al. 2001).

### Reaction phenotyping assays with recombinant P450s

Thirteen cDNA-expressed human CYP isoforms (CYP1A1, CYP1A2, CYP2A6, CYP2B6, CYP2C8, CYP3A4, CYP2C19, CYP3A5, CYP1B1, CYP2C9, CYP2D6, CYP2E1 and CYP4F2) were used to identify the isoform(s) involved in the biotransformation of toddalolactone. Toddalolactone (10 μM) was incubated with each recombinant CYP (15 nM) for 60 min at 37 °C and metabolites were identified by HPLC.

### Chemical inhibition assays

Specific inhibitors for P450 isoforms were used to explore the contribution of CYP isoforms to the biotransformation of toddalolactone. The specific inhibitors for CYP2C9, CYP1A2, CYP2C19, CYP2D6, CYP2E1 and CYP3A were sulphaphenazole (10 μM), furafylline (10 μM), omeprazole (20 μM), quinidine (10 μM), clomethiazole (50 μM) and ketoconazole (1 μM) (Shi et al. 2019). Clobetasol propionate inhibited CYP3A5 with an IC50 of 0.32 μM (Wang et al. 2021). At 1.8 μM, clobetasol propionate inhibited CYP3A5 by 90% (Wright et al. 2020). We chose clobetasol propionate (1.8 μM) as the selective inhibitor of CYP3A5. In brief, toddalolactone was incubated with HLMs and an NADPH-generating system in the presence and absence (control) of the aforementioned specific inhibitors.

### Molecular docking

For further exploring CYP1A1- and CYP3A5-mediated toddaloactone metabolism, molecular docking was performed from the aspect of recognition and binding between toddaloactone and two P450 enzymes. The Schrodinger package was used to prepare proteins and ligands. A glide was used for molecular docking. Schrodinger Maestro and PyMOL were used for graphic display. The X-ray structures of CYP 1A1 (PDB code 4I8V) and 3A5 (PDB code 5VEU) were obtained from the RCSB Protein Databank (http://rcsb.org/). The toddalolactone structure was built with Marvin Sketch and converted into a three-dimensional structure through LigPrep Wizard within the Schrodinger package. Flexible torsions in the ligands were assigned, and all dihedral angles were allowed to rotate freely. A grid box defined with a 15 Å radius around the native ligand was generated in the allosteric site. Toddalolactone was docked into the defined binding pockets of CYP1A1 and 3A5. Top-ranking docked conformations were selected from all docking conformations based on docking scores and binding modes. Finally, the protein-ligand complexes of chosen conformations were used to elucidate the protein-ligand interactions, which were produced using Maestro and PyMOL.

### Molecular dynamics simulations

Molecular dynamics (MD) simulations for the complexes of CYP1A1-toddalolactone and CYP3A5-toddalolactone were performed from initial conformations to characterize the modelled structures and probe the catalytic process. The crystal structure of toddalolactone complexed with CYP1A1 and CYP3A5 was visualized in the PDB IDs 4I8V and 5VEU, respectively. The initial structure for the CYP-toddalolactone system was obtained from molecular docking methods, as described in the previous section. The models were first solvated in the SPC solvent model. Solvated systems were loaded into the workspace by using the MD panel. Each system was simulated for 200 ns with 200-ps trajectory recording intervals. The system energy was set to 1.2 and equilibrated in the NPT ensemble. Simulations ran at a target temperature of 300.0 K and a target pressure of 1.01325 bar. The option to relax model systems before simulations were selected.

### Western blot analysis

Hep G2 cells were seeded into 100 mm Petri dishes and treated with different concentrations of toddalolactone (0, 10 and 50 µmol/L) for 24 h. Total protein was extracted using RIPA lysis buffer containing proteinase inhibitor (BestBio, Shanghai, China). After the protein concentration was measured using a bicinchoninic acid (BCA) assay kit (Beyotime, Shanghai, China), samples were electrophoretically transferred to polyvinylidene difluoride (PVDF) membranes. After blocking with TTBS (0.5% Tween 20, 10 mM Tris-HCl, pH 7.5, 150 mM NaCl) containing 5% non-fat milk for 1 h at room temperature, the membrane was incubated with antibodies against β-Actin, CYP1A1, and CYP3A5 overnight at 4 °C. Then, membranes were washed with TBS (10 mM Tris-HCl, pH 7.5, 150 mM NaCl) containing 0.05% Tween 20 three times and further incubated with HRP-conjugated secondary antibodies against rabbit (for β-actin, CYP1A1 and CYP3A5) for 1 h at room temperature.

### Pharmacokinetics study

Six male Sprague–Dawley rats weighing 200 ± 10 g were obtained from the Laboratory Animal Center of Jinzhou Medical University. Approval of the study protocols was obtained from the Institutional Animal Ethics Committee before the commencement of the studies. Toddalolactone was administered intravenously into the tail vein at a dose of 10 mg/kg, and 0.5 mL of blood was collected through orbital venous plexus at 0, 0.25, 0.5, 0.75, 1, 2, 4, 6 and 8 h after toddalolactone administration and centrifuged at 2000 *g* for 5 min at 4 °C to obtain the plasma samples. The plasma (0.1 mL) was mixed with 200 μL acetonitrile, the mixture was vortexed for 30 s and then centrifuged at 20,000 *g* for 20 min and the supernatant was collected and analyzed by HPLC.

### Analysis of metabolites by LC-MS/MS

After toddalolactone was incubated with MIMs and HLMs in the phase I (CYPs) system, identification of metabolites was performed on an LC-MS/MS. Mass detection was obtained in positive ion mode from *m*/*z* 100 to 1000. The LC-MS/MS system was coupled with the Shimadzu LC-20A HPLC system equipped with a C18 column (150 mm × 2.0 mm, 5 µm, Shimadzu, Kyoto, Japan).

The mobile phase consisted of water that included 0.1% formic acid (A) and acetonitrile (B) with the elution gradient are as follows: 0.01–2 min, 5% B; 2–10 min, 5–95% B; 10–12 min, 95% B; 12–12.5 min, 95–5% B; and 12.5–15 min, 5% B. The flow rate was 0.4 mL/min, the column temperature was maintained at 40 °C and the injection volume was 10 μL.

The MS conditions were as follows: ion source gas 1 and gas 2 were 50 psi, curtain gas was set at 35 psi, ion spray voltage was set at ±5000 kV, ion source temperature was set at 500 °C, collision energy was set at ±35 eV, and declustering potential was set at 80 V. The declustering potential and collision energies were 100 V and ±40 V, respectively.

### Analytical instruments and conditions

The peak areas of toddalolactone and its metabolites were detected using a HPLC system (Shimadzu, Kyoto, Japan). The HPLC system consisted of two LC-20AB pumps, a SIL-20A auto-injector, a CTO-20AB column oven, and an SPD-20A ultraviolet (UV) detector. The separation of toddalolactone and its metabolites was achieved using a Hypersil BDS C18 column (Dalian Elite Analytical Instruments Co., Dalian, China; 4.6 × 150 mm, 5 μm). The mobile phases consisted of acetonitrile (solvent A) and 0.08% formic acid (solvent B) with the following gradient program: 0.00–13.00 min, 45–55% B; 13.00–13.30 min, 55–90% B; 13.30–18.30 min, 90% B; 18.30–19.00 min, 90–45% B; 19.00–23.00 min, 45% B. The flow rate was 1 mL/min, and samples were detected at 328 nm.

## Statistical analysis

The data collected were statistically evaluated and analyzed with SPSS Ver.18.0 (Chicago, IL). All data are expressed as mean ± SD. Comparisons between the two unpaired groups were performed using Student’s *t*-test. Differences between multiple groups were tested by one-way analysis of variance. Two-tailed *p* < 0.05 was considered to indicate a statistically significant difference.

## Results

### Metabolic profiling of toddalolactone in liver microsomes of various species

**Figure 2.**
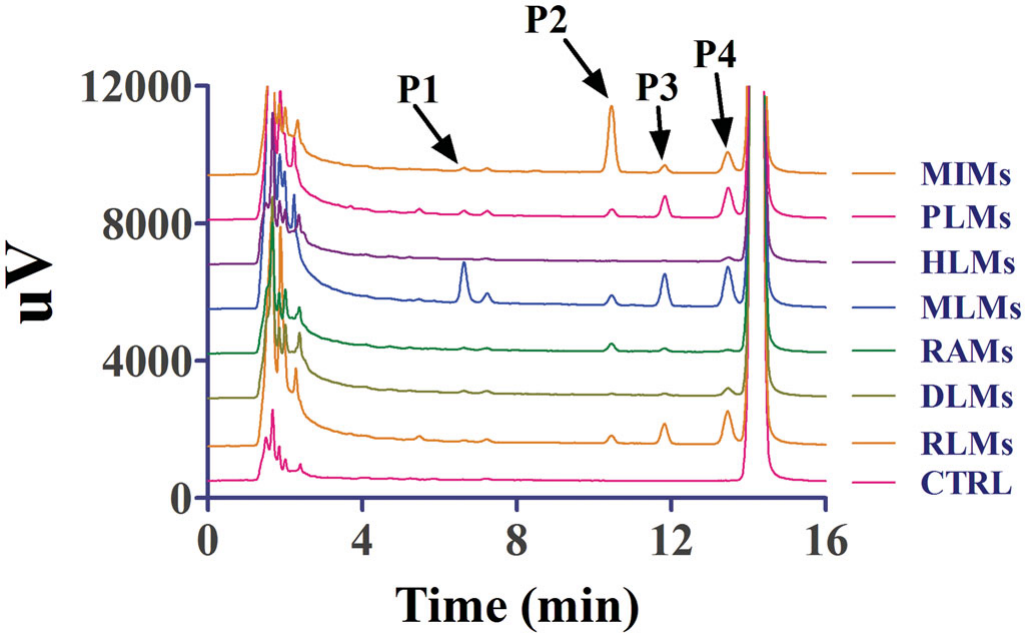
Representative LC profiles of toddalolactone and its metabolites produced in HLMs, RLMs, PLMs, RAMs, MLMs, DLMs and MIMs. Toddalolactone (20 μM) was incubated with an NADPH-dependent system at 37 °C for 60 min.

**Figure 3.**
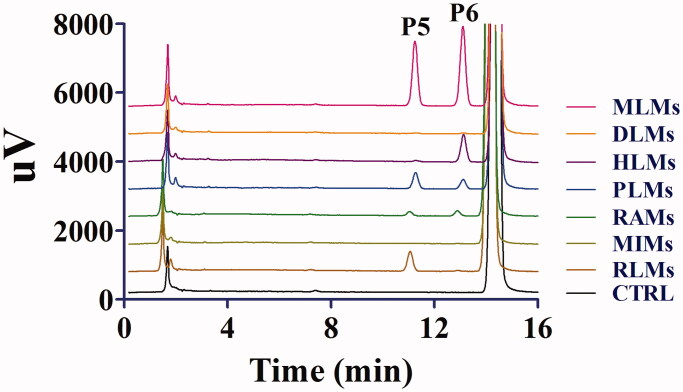
Representative HPLC profiles of toddalolactone and its metabolites in MLMs, DLMs, HLMs, PLMs, RAMs, MIMs and RLMs. Toddalolactone (20 μM) was incubated with a UDPGA-dependent system at 37 °C for 60 min.

After the incubation of toddalolactone with different liver microsomes with an NADPH-generating system, four new peaks were eluted, at 6.6 min (P1), 10.4 min (P2), 11.8 min (P3) and 13.4 min (P4) (Figure 2). After toddalolactone was incubated with different liver microsomes in the UDPGA-generating system, two new peaks were detected at 11.2 min (P5) and 13.1 min (P6), respectively (Figure 3). The peak area percentages of each metabolite in different microsomes are reported in Table 1.

**Table 1.** Peak area percentages of each metabolite in different microsomes.

Species	Peak area (%)					
	P1 (6.6 min)	P2 (10.4 min)	P3 (11.8 min)	P4 (13.4 min)	P5 (11.2 min)	P6 (13.1 min)
HLMs	N.D.	N.D.	1.20	9.08	N.D.	34.75
DLMs	6.09	1.60	5.65	17.06	N.D.	N.D.
PLMs	9.79	10.76	65.23	70.28	25.65	12.07
MLMs	100.00	14.23	100.00	100.00	100.00	100.00
RAMs	5.69	11.12	10.12	4.08	6.57	6.18
RLMs	3.16	10.67	63.20	83.30	31.68	N.D.
MIMs	6.44	100.00	22.38	47.58	N.D.	N.D.

The species with largest metabolite peak is set as 100% and the other species as %. N.D.: No detect.

### Kinetic analyses

**Figure 4.**
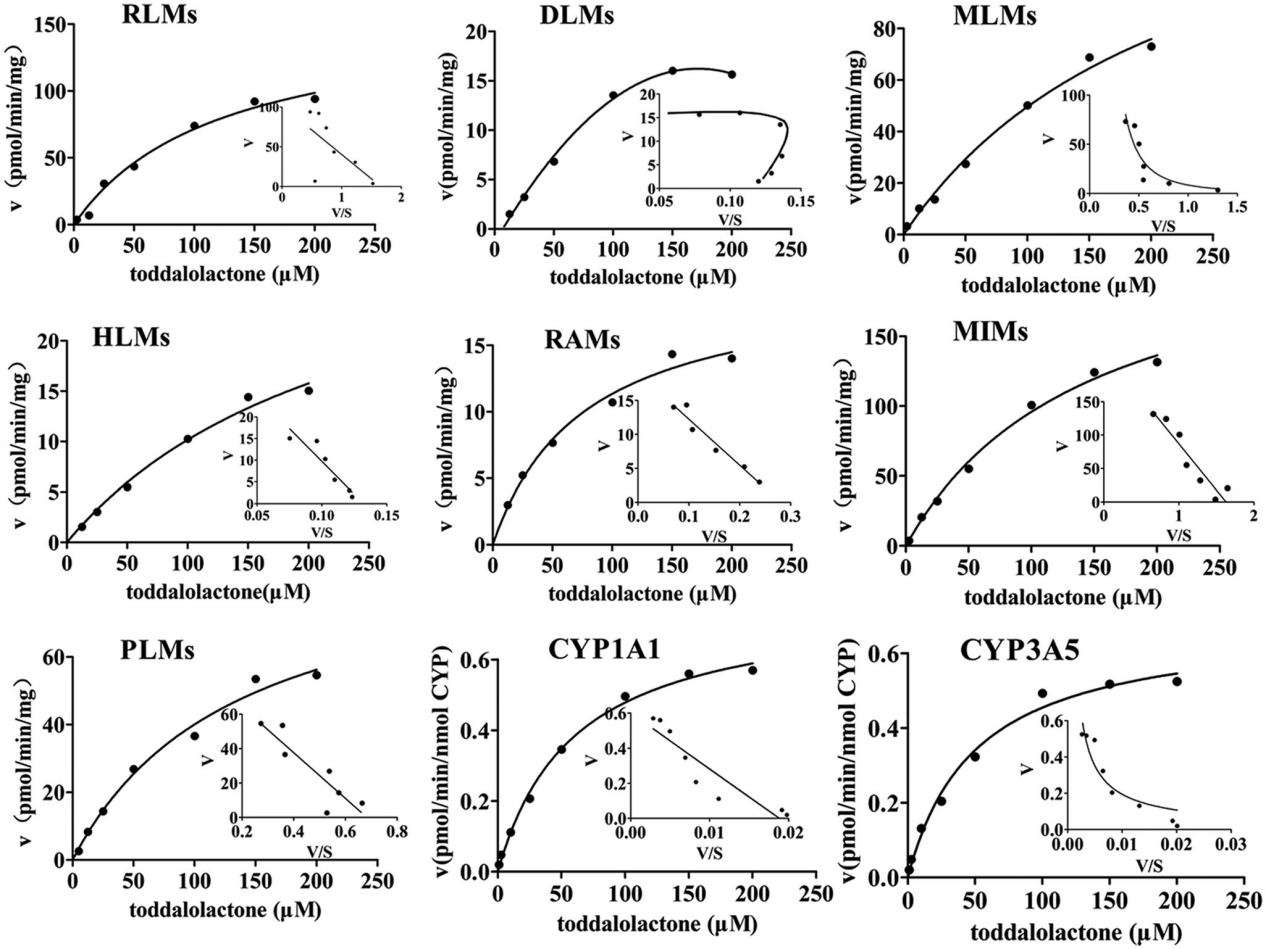
Kinetics of toddalolactone metabolism in RLMs, DLMs, MLMs, HLMs, RAMs, MIMs, PLMs, CYP1A1 and CYP3A5 in the presence of an NADPH system. The Eadie–Hofstee plots (V–V/S plot) are shown as an inset. Data represent the mean of triplicate determinations.

**Figure 5.**
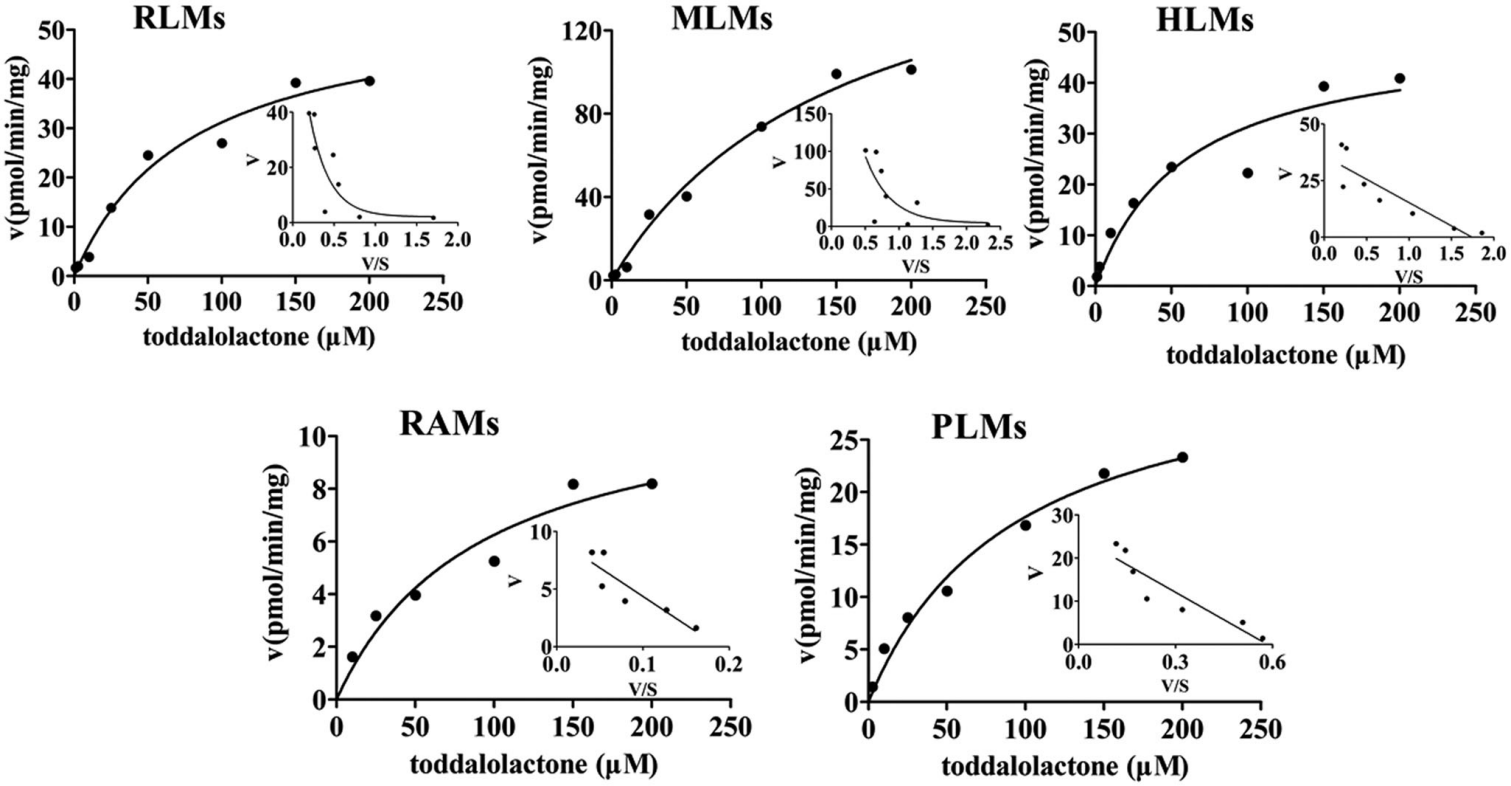
Kinetics of toddalolactone glucuronidation in RLMs, MLMs, HLMs, RAMs and PLMs. The Eadie–Hofstee plots (V–V/S plot) are shown as the inset. Data points represent the mean of triplicate determinations.

The enzyme kinetics in oxidative metabolism and glucuronidation of toddalolactone were studied, and preliminary experiments were performed to ensure that metabolites were formed in the linear range of both reaction time and microsomal protein concentration. The metabolite with the largest peak area was used to calculate the kinetic parameter values (*K*_m_, *V*_max_ and CL_int_). Based on the results of toddalolactone metabolism in different species, the kinetic parameter values in RLMs, DLMs, MLMs, HLMs, RAMs, MIMs, PLMs, CYP1A1 and CYP3A5 were calculated using P4, P4, P4, P4, P2, P2, P4, P4 and P4 data in oxidative metabolism, respectively. In the glucuronidation reaction, the kinetic parameter values in RLMs, MLMs, HLMs, RAMs and PLMs were calculated using P5, P6, P6, P6 and P5 data, respectively. The metabolism profiles of toddalolactone (1–200 μM) in CYP-mediated and UGT-mediated reactions are shown in Figures 4 and 5, respectively. The reaction velocities exhibited major concentration-dependent characteristics. The kinetic parameters for the species in CYP-mediated and UGT-mediated reactions are summarized in Tables 2 and 3.

**Table 2.** Kinetic parameters of metabolite production from toddalolactone in P450-catalyzed drug oxidation reactions.

Kinetic parameters	RLMs^a^	DLMs^a^	MLMs^a^	HLMs^a^	RAMs^a^	MOMs^a^	PLMs^a^	CYP1A1^b^	CYP3A5^b^
*V*_max_	158.26 ± 20.52	29.76 ± 7.27	161.4 ± 25.73	35.07 ± 7.16	20.03 ± 1.72	235.91 ± 22.75	94.24 ± 11.61	0.76 ± 0.03	0.68 ± 0.04
*K*_m_	121.13 ± 31.89	148.93 ± 38.37	255.67 ± 58.29	244.63 ± 78.99	76.46 ± 16.02	146.32 ± 26.62	135.36 ± 32.39	60.12 ± 6.67	50.34 ± 8.62
CL_int_	1.31	0.19	0.63	0.14	0.26	1.61	0.69	0.011	0.013

^a^*K*_m_, *V*_max_, and CL_int_ are expressed in units of µM, pmol/min/mg protein, and µL/min/mg protein, respectively.

^b^*K*_m_, *V*_max_, and CL_int_ are expressed in units of µM, pmol/min/nmol CYP, and µL/min/nmol CYP, respectively.

**Table 3.** Kinetic parameters of metabolite production from toddalolactone in glucuronide metabolism.

Kinetic parameters	RLMs	MLMs	HLMs	RAMs	PLMs
*V*_max_	55.89 ± 6.61	187.53 ± 27.77	50.16 ± 8.75	11.98 ± 2.19	34.12 ± 3.62
*K*_m_	79.11 ± 22.56	154.51 ± 42.46	66.33 ± 20.46	91.46 ± 28.22	93.28 ± 22.35
CL_int_	0.69	1.21	0.75	0.12	0.36

*K*_m_, *V*_max_, and CL_int_ are expressed in units of µM, pmol/min/mg protein, and µL/min/mg protein, respectively.

### Prediction of *in vivo* hepatic clearance in rats, dogs, and humans

The kinetic parameters of P1, P2, P3, P4, P5 and P6 generated in RLMs, HLMs and DLMs were used to calculate *in vivo* hepatic clearance (CL_H_); the results were 0.47, 5.25 and 1.12 mL/(min/kg body weight) for dogs, humans and rats, respectively. The CL_H_ versus hepatic blood flow (QH) for RLMs, HLMs and DLMs was 1.52%, 8.68% and 5.41%, respectively.

### Metabolic stability assessment with different liver microsomes

**Figure 6.**
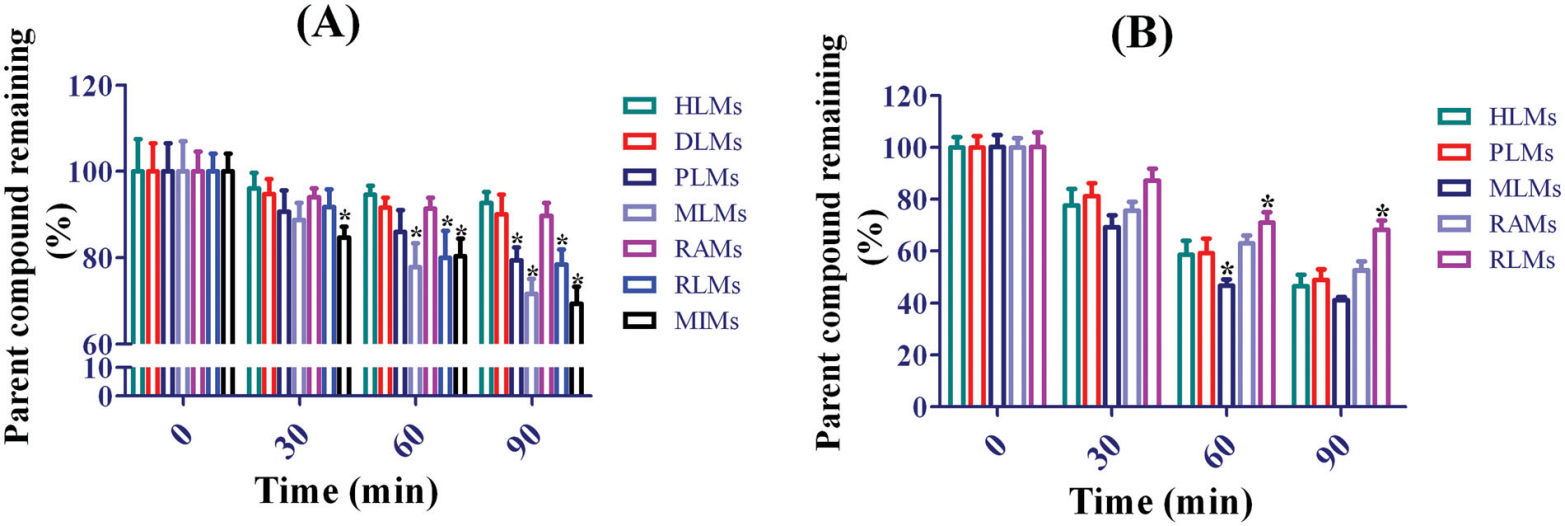
Metabolic stability profiles of toddalolactone in HLMs, DLMs, PLMs, MLMs, RAMs, RLMs and MIMs for oxidative metabolism (A) and glucuronidation of toddalolactone (B). Data represent the mean ± SD. *N* = 3. *p < 0.05 versus HLMs at each time-point (0, 30, 60 and 90 min).

The metabolic stabilities of toddalolactone in CYP-mediated and UGT-mediated reactions were determined using different liver microsomes. The results are shown in Figure 6. The half-life value (*T*_1/2_) of toddalolactone in the CYP incubation system was 673 ± 36, 494 ± 42, 307 ± 28, 245 ± 19, 407 ± 31, 382 ± 17 and 235 ± 21 min for HLMs, DLMs, PLMs, MLMs, RAMs, RLMs and MIMs, respectively. The *T*_1/2_ value of toddalolactone in the UGT incubation system was 83 ± 8.2, 85 ± 4.3, 66 ± 7.6, 124 ± 8.3 and 129 ± 11.8 min for HLMs, PLMs, MLMs, RAMs and RLMs, respectively.

### Assays with recombinant human CYP isoforms

**Figure 7.**
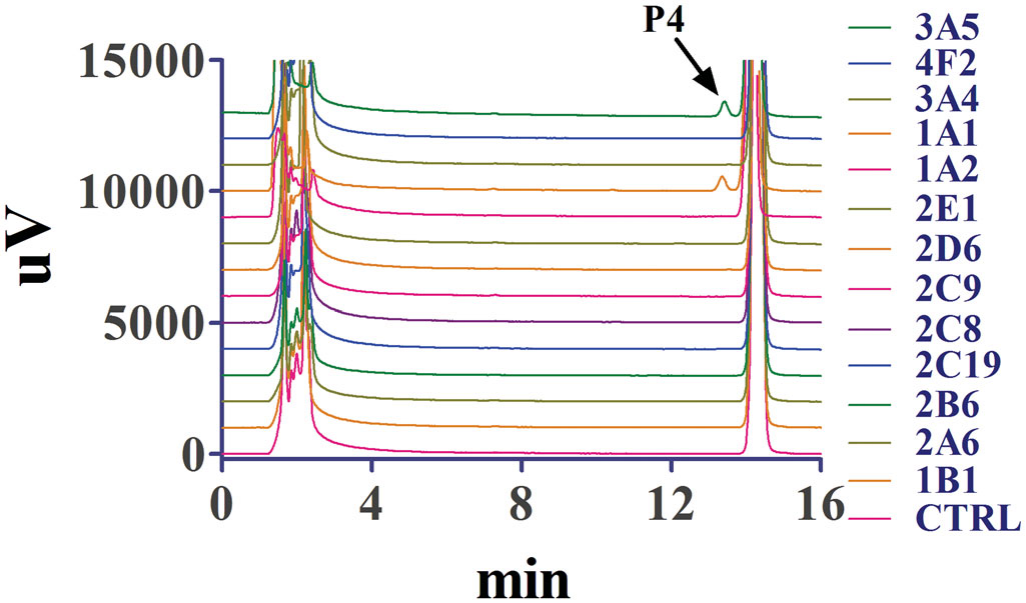
Representative chromatogram of toddalolactone and its metabolites incubated with recombinant human cytochrome P450s. Toddalolactone (20 μM) was incubated with each P450 isoform (15 nM) at 37 °C for 60 min.

Thirteen recombinant human cytochrome P450 (rhCYP) isoforms were used to test their activities in the biotransformation of toddalolactone so as to evaluate the CYP isoforms involved in the metabolism of toddalolactone in the human body. As shown in Figure 7, toddalolactone metabolism was predominantly catalyzed by CYP3A5 and CYP1A1. P4 was the main metabolite catalyzed by two rhCYP isoforms, whereas other rhCYP isoforms displayed very limited or even no catalytic activity in this biotransformation. All the rhCYP isoforms showed a limited effect on the formation of P1–P3.

### Chemical inhibition assays

**Figure 8.**
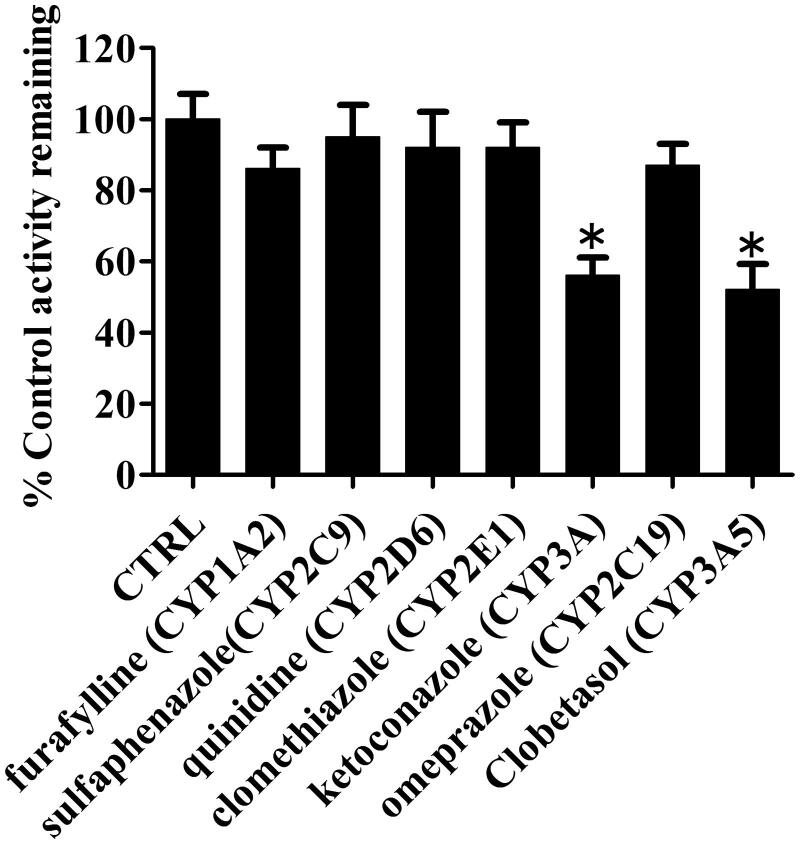
Effects of selective P450 inhibitors on the formation of metabolite (P4) in HLMs. Results are shown as mean ± SD of at least three separate assays.

Chemical inhibition assays were carried out using selective inhibitors of major P450 isoforms to further verify the key P450 isoform(s) involved in the formation of P4 in HLMs. Among the seven selected inhibitors, ketoconazole (a potent inhibitor of CYP3A) and clobetasol propionate **(**a potent inhibitor of CYP3A5**)** substantially inhibited ∼40% formation of P4 compared with other CYP isoform inhibitors (Figure 8). Other inhibitors showed no significant inhibitory effects on P4 formation.

### Docking simulations

**Figure 9.**
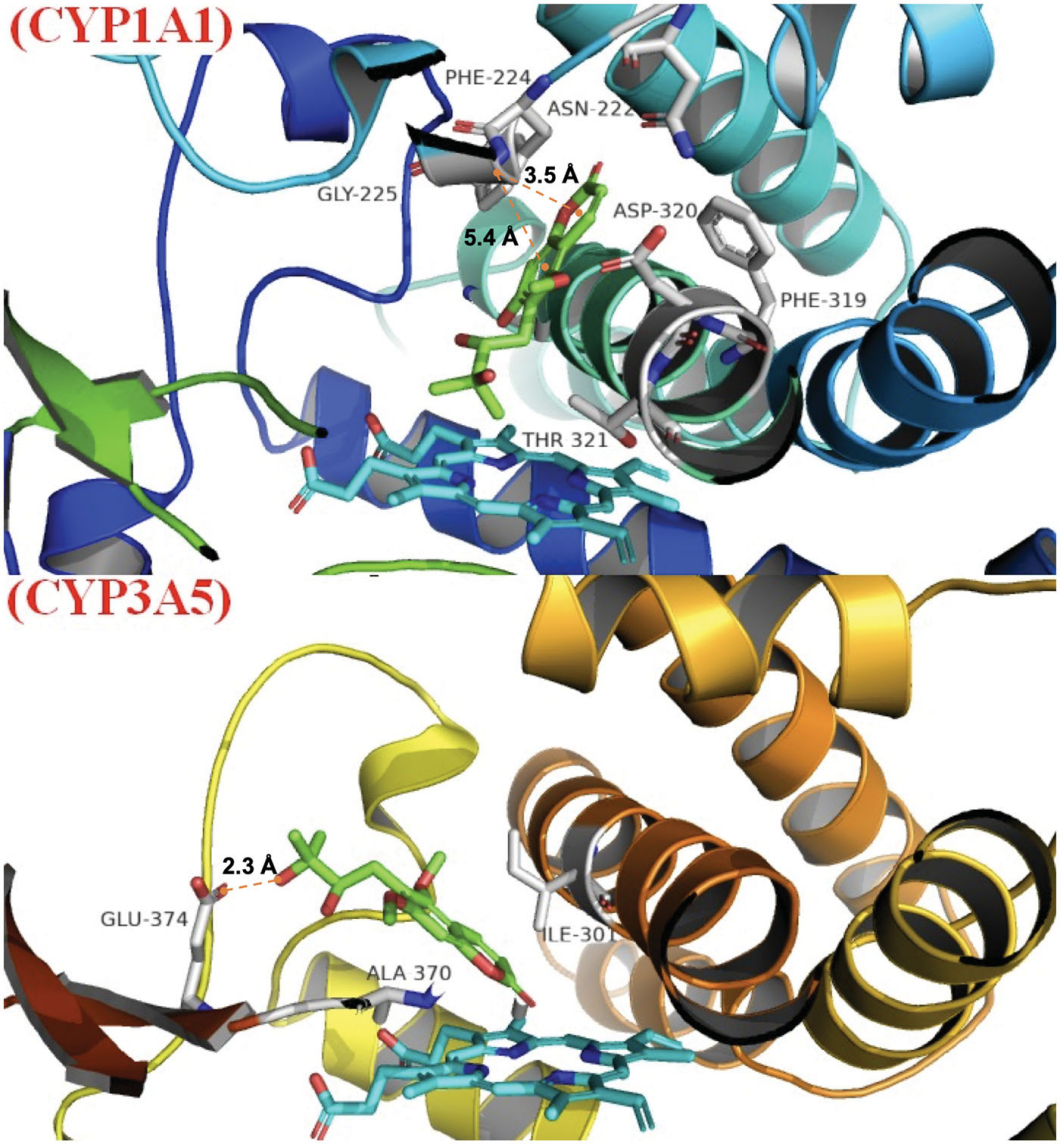
Binding mode of toddalolactone with CYP 1A1 and CYP3A5. CYP protein is shown as a ribbon, Haem is shown as the blue stick, and toddalolactone is displayed as the green stick.

The docking score for toddalolactone was −7.158 kcal/mol with CYP1A1 and −5.473 kcal/mol with CYP3A5. Toddalolactone is bound within the binding pockets of CYP1A1 and 3A5, surrounded by amino acids ASN222, PHE224, GLY225, PHE319, ASP320 and THR321 of CYP1A1 (Figure 9(A)) and amino acids ILE301, ALA370 and GLU374 of CYP3A5 (Figure 9(B)). Pi–pi (π–π) stacking was observed between toddalolactone and PHE224 within the CYP1A1 pocket, and a hydrogen bond was detected between toddalolactone and GLU374 within the CYP3A5 pocket. Toddalolactone interacted with Phe224 of CYP1A1 through π–π stacking with distances of 3.5 Å and 5.4 Å, and interacted with Glu374 of CYP3A5 through an H-bond with a distance of 2.3 Å.

### MD simulations on CYP1A1/CYP3A5–toddalolactone complexes

**Figure 10.**
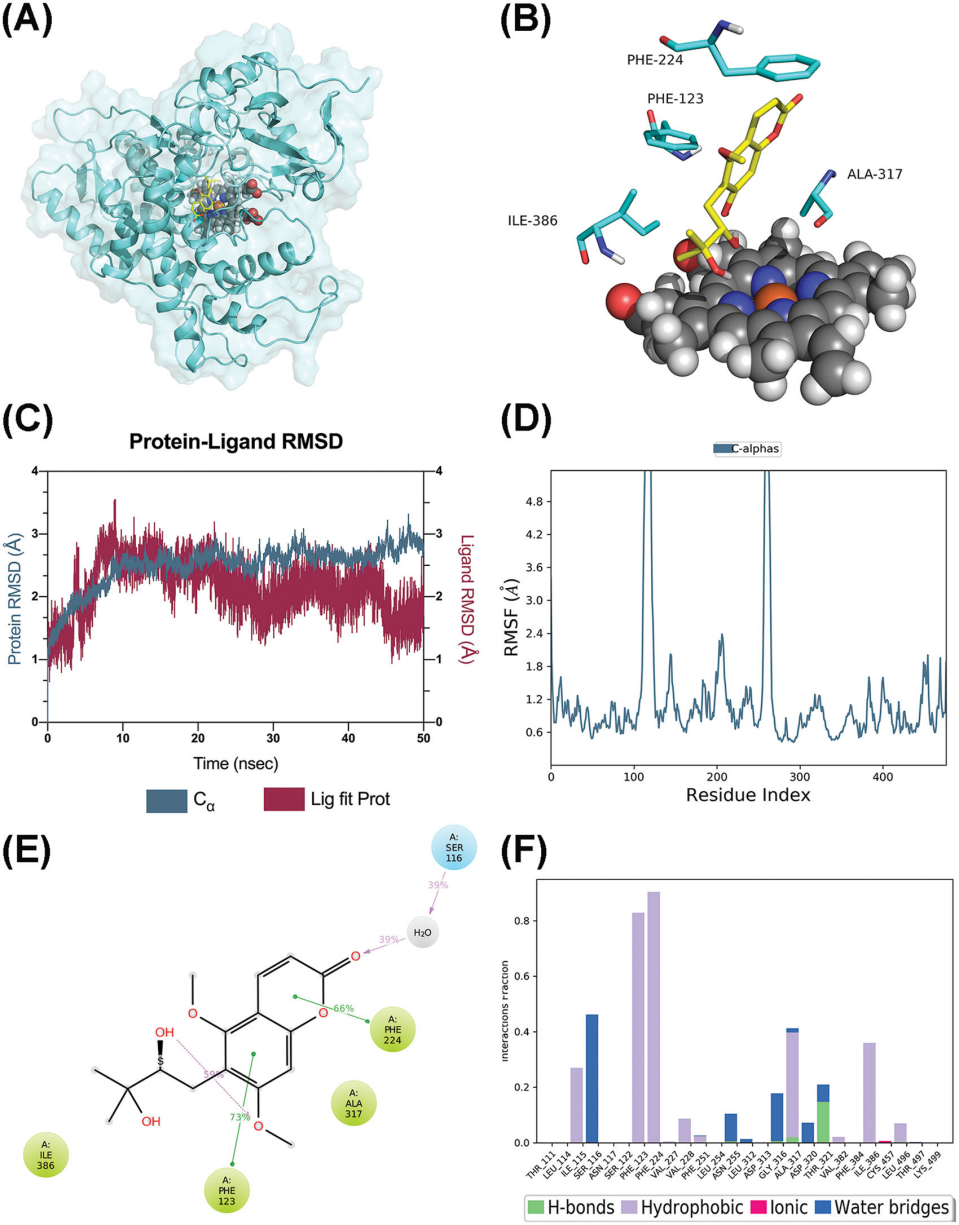
Molecular dynamics analysis of the P450 1A1/toddalolactone complex. (A) The P450 1A1 is shown in the surface representation (PDB code: 4I8V). (B) A 3D view of the binding pose. The major amino acids located at the active site are shown. (C) RMSD plot over a 50 ns simulation. (D) RMSF curve during the 50 ns MD simulation. (E) A 2D interaction diagram of P450 1A1 with toddalolactone. Hydrogen bond interactions are rendered as purple arrows, and pi–pi stacking interactions are denoted by green solid lines. (F) Interaction fraction of CYP 1A1 key amino acids from the 50 ns MD simulation.

Both CYP 1A1 and CYP3A5 composited with the toddalolactone were submitted for a 50 ns period of molecular dynamics (MD) simulations to evaluate the binding patterns revealed from the molecular docking study. P450 1A1 formed a stable complex with toddalolactone (Figure 10(A)), with major interactions with PHE123, PHE224, ALA317 and ILE386 (Figure 10(B)). Aftera short period of fluctuation in the first 10 ns, the average root-mean-square deviation (RMSD) value stabilized at 2.8 Å (Figure 10(C)), confirming that CYP1A1 complexes reached a steady state in the dynamic simulations. The analysis of the root-mean-square fluctuation (RMSF) of the protein showed that the RMSF values of the key amino acids PHE123 and PHE224 were low, indicating that they exerted a stable hydrophobic effect with toddalolactone (Figure 10(D)). PHE123 (73%), PHE224 (66%), ALA317 (37%) and ILE386 (35%) mainly contributed to the hydrophobic pocket. Among these, PHE123 and PHE224 were involved in the formation of π–π stacking interactions with the aromatic system of toddalolactone. In addition, SER116 formed a water bridge with the carbonyl group of the coumarin moiety for 39% of the whole MD period (Figure 10(E,F)).

**Figure 11.**
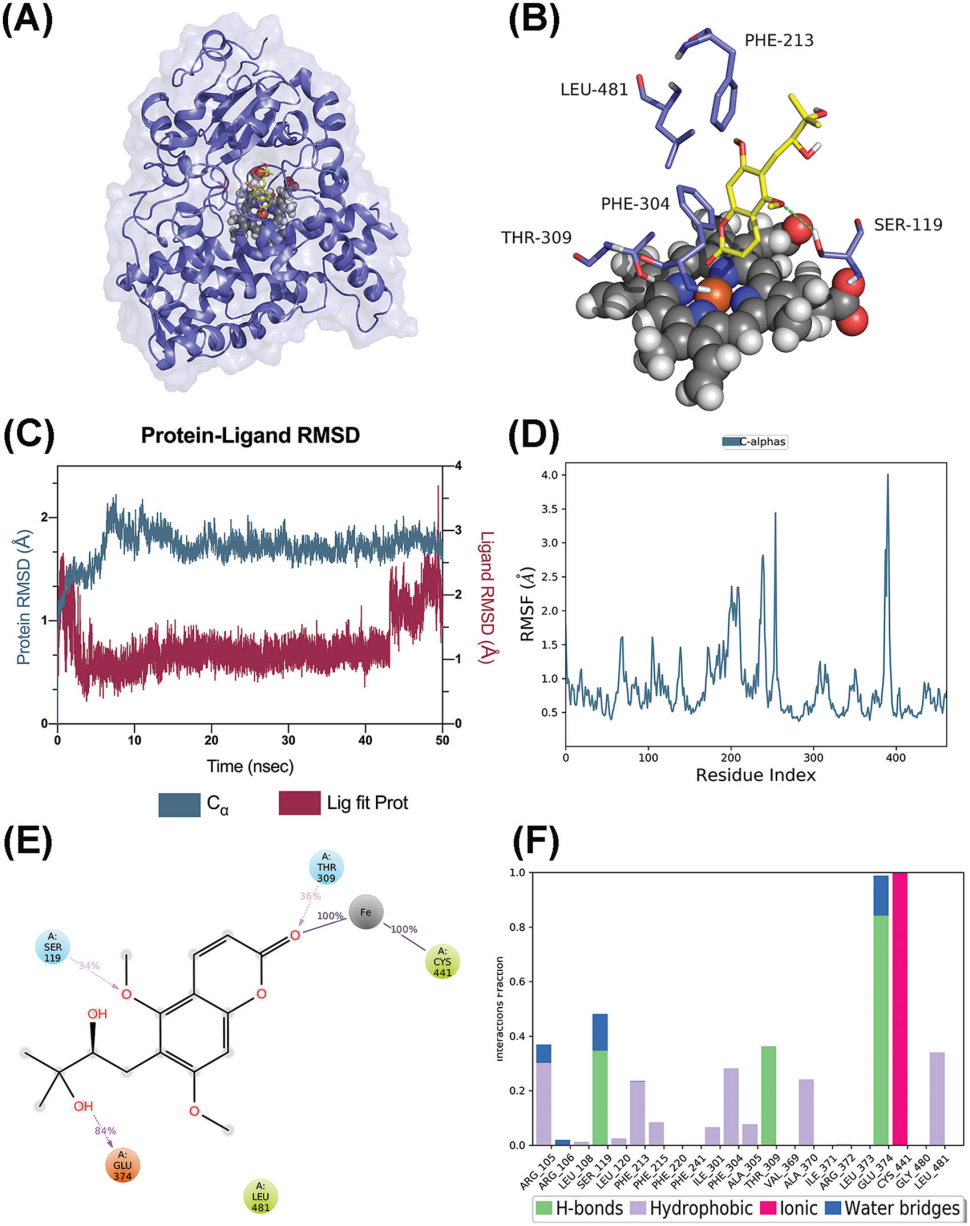
Molecular dynamics analysis of the P450 3A5/toddalolactone complex. (A) P450 3A5 is shown in the surface representation (PDB code: 5VEU). (B) A 3D view of the binding pose. The major amino acids located at the active site are shown. (C) RMSD plot over the 50 ns simulation. (D) RMSF curve during the 50 ns MD simulation. (E) A 2D interaction diagram of P450 3A5 with toddalolactone. Hydrogen bond interactions are rendered as purple arrows, and pi-pi stacking interactions are denoted by green solid lines. (F) Interaction fraction of CYP3A5 key amino acids from the 50 ns MD simulation.

P450 3A5 formed a stable complex with toddalolactone (Figure 11(A)), and extensive binding patterns were observed involving GLU374, SER119, PHE213, PHE304, THR309, LEU481 and haem, which mainly interacted with toddalolactone (Figure 11(B)). After a short period of fluctuation in the first 10 ns, the average RMSD value stabilized under 2.7 Å (Figure 11(C)), indicating that the CYP3A5 complex reached a steady state during the dynamic simulations. In terms of RMSF, the major amino acids located at the binding site barely represented any RMSF fluctuations, which were observed to be stable and consistent during the simulation time (Figure 11(D)). The carbonyl group of the coumarin moiety formed a stable coordination moiety attached to CYS441 of CYP3A5 through the iron ion. GLU374 (84%), THR309 (36%) and SER119 (34%) formed hydrogen bonds with toddalolactone. GLU374 acted as a hydrogen bond acceptor to interact with the hydroxyl group of toddalolactone, while THR309 and SER119 acted as hydrogen bond donors to bond with the carbonyl and ether groups, respectively. In addition, LEU481 participated in the hydrophobic effect that occurred during more than 30% of the simulation (Figure 11(E, F)). Together, these data supported the previous molecular docking results that toddalolactone was metabolized by CYP1A1 and CYP3A5 through stable receptor-ligand interactions, and PHE224 in CYP1A1 and GLU374 in CYP3A5 are major players for high catalytic efficiency towards toddalolactone.

### Impact of toddalolactone on CYP1A1 and CYP3A5 protein expression in HepG2 cells

**Figure 12.**
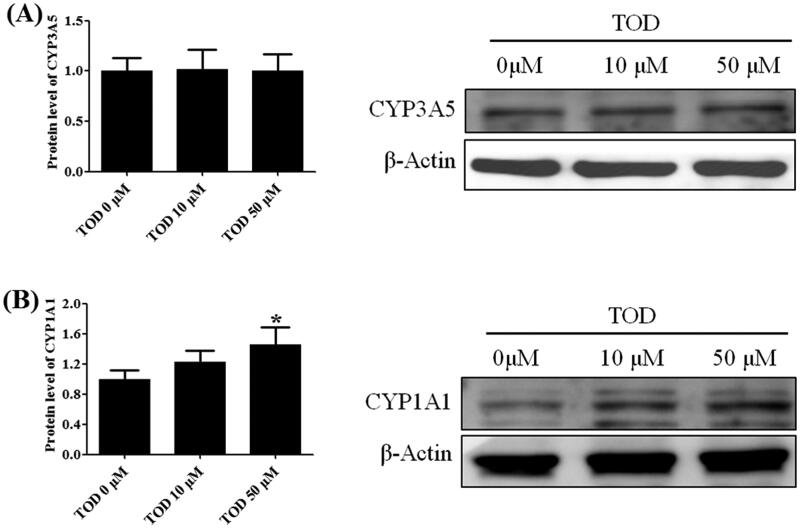
Effect of toddalolactone (TOD) on CYP3A5 (A) and CYP1A1 (B) protein levels in HepG2 cells at various concentrations (0 μM, 10 μM and 50 μM). DMSO (0.1%) in the absence of toddalolactone was used as the control group. Results are presented as fold-induction relative to control. Values are means ± SD (*n* = 4) and the significant difference compared with the control group, *p < 0.05.

After 24-h treatment with different concentrations of toddalolactone (0, 10 and 50 μM), the protein expression of CYP3A5 (Figure 12(A)) in HepG2 cells did not show any significant change. However, the protein expression of CYP1A1 (Figure 12(B)) was gradually upregulated. For the highest toddalolactone concentration of 50 μM, induction of CYP1A1 protein expression was approximately 50% higher than that of the control (0 μM, *p* < 0.05).

### Pharmacokinetics

**Figure 13.**
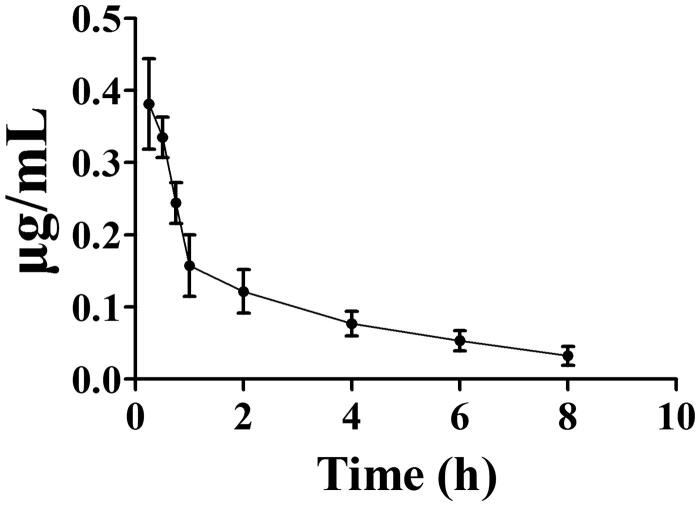
The plasma concentration profiles after intravenous administration of toddalolactone at a single dose of 10 mg/kg. Data represent the mean ± SD of six rats.

After intravenous administration of toddalolactone, blood samples were collected and the concentration of toddalolactone in plasma was determined. The mean plasma concentrations of toddalolactone versus time profiles are depicted in Figure 13. The pharmacokinetic (PK) parameters such as AUC_0–t_, *t*_1/2_, *T*_max_ and *C*_max_ were calculated by a noncompartmental method using the DAS 3.1 software package (Jiangxi University of Traditional Chinese Medicine, Nanchang, China). The mean *C*_max_ value for toddalolactone was 0.42 μg/mL, and *T*_max_ occurred at 0.25 h post-dosing. The elimination *t*_1/2_ was estimated as 1.05 h, and the AUC_0–t_ was found to be 0.46 μg/mL/h.

### Structural identification of the metabolites of toddalolactone

**Figure 14.**
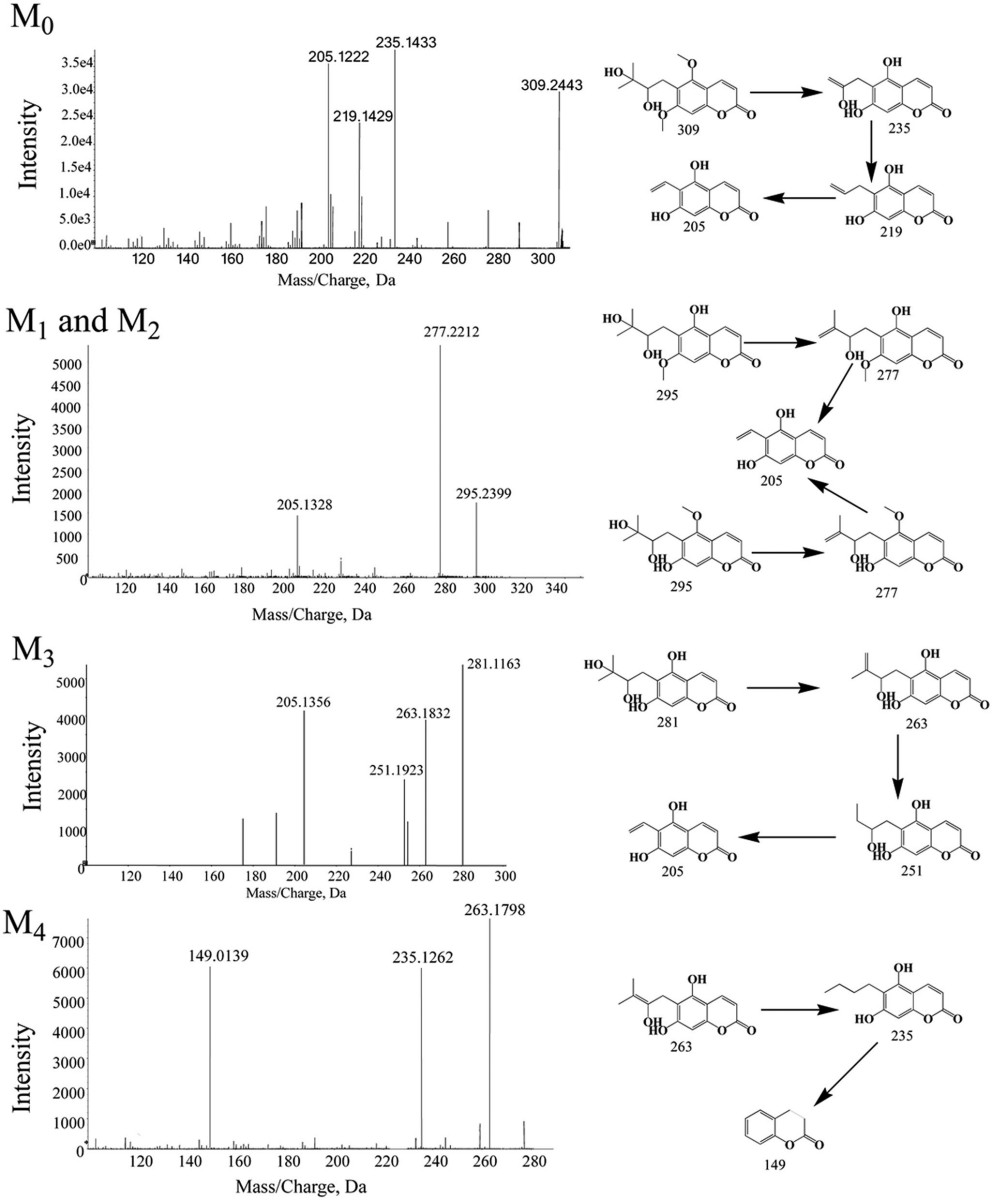
Mass spectrum (MS/MS) of toddalolactone and its metabolites.

According to the HPLC profiles from different species (shown in Figure 2), we found that mice produced the majority of the new peaks (P1–P4) compared with other species in phase I (CYPs) system. In this study, LC-TPF-MS/MS was employed to identify the metabolites after incubation with the liver microsomes from mice and humans containing the NADPH-generating system. Four metabolites of toddalolactone were identified based on a direct comparison with the mass spectra of the blank samples. The MS/MS spectra of toddalolactone and its metabolites are shown in Figure 14.

#### Parent drug (M0)

Toddalolactone, with a formula of C_6_H_20_O_6_ (*m*/*z* 309.2443), was eluted at 6.8 min. Toddalolactone exhibited three characteristic fragment ions at *m*/*z* 235.1433, 219.1429 and 205.1222. The fragment ion at *m*/*z* 235.1433 was formed by the loss of three methyl groups from the parent molecule at C-17, C-19 and C-14(15), which produced further fragment ions at *m*/*z* 219.1429 and 205.1222.

#### M1 and M2

M1 (*R*t = 6.42 min) and M2 (*R*t = 6.21 min) were isomers with the same molecular formula of C_15_H_19_O_6_ (*m*/*z* 295) and different *R*t. Their *m*/*z* was 14 Da lower than that of toddalolactone, indicating that they were the hydrolytic metabolites of the parent drug. The hydrolysis reaction occurred at O-16 (M1) or O-18 (M2). M1 and M2 had the same fragment ions at *m*/*z* 277 and 205.

#### M3 and M4

Metabolite M3 with [M-H]^+^ at *m*/*z* 281.1163 was 14 Da lower than M1 or M2 and was identified as the hydrolytic product of M1 or M2 according to the MS/MS spectra. The side chain linked at C-7 in ring (a) underwent a series of cleavage reactions and generated product ions at *m*/*z* 263.1832, 251.1923 and 205.1356. The molecular formula of M4 was determined as C_14_H_14_O_5_ (*m*/*z* 263.1798) with two major characteristic fragment ions at *m*/*z* 235.1262 and 149.0139. M4 was formed by the loss of one hydroxyl group from M3.

## Discussion

Animal models are often used to predict the metabolic behaviour of new compounds in the human body during the preclinical development of drugs. Therefore, animal species with metabolic characteristics like those of human beings should be first selected. However, significant species-specific differences exist in the expression and function of drug-metabolizing enzymes, including CYPs and UGTs, between humans and animals. For example, UGT1A4 and UGT1A9 are not expressed in rats (Guo et al. 2015). *In vitro* metabolism studies were also used to help rapidly and conveniently screen animal species (Mi et al. 2014). In this study, the species-specific differences in NADPH- and UDPGA-dependent metabolism of toddalolactone were first identified using liver microsomes. The metabolic profiling study revealed that toddalolactone could be metabolized by liver microsomes from all examined species, including humans, monkeys, rabbits, dogs, minipigs, rats, and mice; six metabolite peaks (P1–P6) were detected by HPLC. Toddalolactone metabolism exhibited large species-specific differences. Three (P3, P4 and P6), four (P1–P4), six (P1–P6), six (P1–P6), six (P1–P6), five (P1–P5) and four (P1–P4) toddalolactone metabolites were detected in HLMs, DLMs, PLMs, MLMs, RAMs, RLMs and MIMs, respectively. Monkeys exhibited the greatest metabolic capacity compared with other species. The data in Table 1 show that the peak area percentage of P1, P3, P4, P5 and P6 in MLMs was 76.23%, 37.36%, 30.18%, 60.01% and 65.35%, respectively, which were higher than those in other liver microsomes. Humans showed a weaker metabolic capacity for toddalolactone compared with other species in the phase I (CYPs) reaction system. Due to the two hydroxyl groups of toddalolactone, it is likely that glucuronidation is an important pathway for toddalolactone clearance in humans. After toddalolactone was incubated with HLMs (UGT incubation system), the main metabolite (P6) of toddalolactone was observed. The structures of the metabolites were characterized by LC-MS/MS after toddalolactone was incubated with HLMs and MIMs. According to the MS/MS spectra, these metabolites were primarily formed by hydrolysis. Since the catalytic efficiency and affinity of substrate(s) are fundamental for evaluating species-specific differences towards given biotransformation (Ma et al. 2017; Wang et al. 2020), the enzyme kinetics for toddalolactone metabolism in liver microsomes from various species were established. Enzymatic kinetic analyses showed that oxidative metabolism profiles of toddalolactone for humans, rats, rabbits, mice and pigs followed the classic Michaelis–Menten kinetics, as evidenced by an Eadie–Hofstee plot, suggesting that toddalolactone was metabolized by the species through only one isozyme or two isozymes with the same *K*_m_ value (Jiang et al. 2020), while such biotransformation in monkeys and dogs obeyed biphasic kinetics and substrate inhibition, respectively. The phase II glucuronidation reaction of toddalolactone in humans, rabbits and pigs followed Michaelis–Menten kinetics, whereas toddalolactone glucuronidation in rats and monkeys obeyed biphasic kinetics. Biphasic kinetics may be observed when a reaction is carried out by more than one enzyme with largely different kinetic properties. P4 formations in recombinant CYP1A1 and CYP3A5 followed Michaelis–Menten and biphasic kinetics, respectively. Biphasic kinetics has also been observed in reactions with a purified enzyme. In this case, the enzyme may have multiple binding sites interacting with the substrate having different *K*_m_ and *V*_max_ values (Seibert and Tracy 2014). For example, the *O*-demethylation of naproxen, which is catalyzed by CYP2C9, displays biphasic kinetics, suggesting that two naproxen molecules may bind to the active site simultaneously and exhibit different kinetic properties (Wei et al. 2007). The CYP3A subfamily has large substrate-binding pockets and can bind to two or more of the same substrate molecules that exhibit homotropic or heterotropic cooperativity (Dai et al. 2019). Substrate inhibition occurs when the concentration of the substrate exceeds a certain value, resulting in a decrease in the metabolic rate (Korzekwa et al. 1998). In this study, monkeys showed the strongest metabolic capacity in both CYP-mediated and UGT-mediated reactions with the largest peak areas for P1, P3, P4, P5 and P6 among the species. The glucuronidation of toddalolactone in humans was greater due to the higher peak areas of metabolites M5 and M6. Toddalolactone contains two alcoholic hydroxyl groups. The glucuronidation reaction may occur through transferring a glucuronic acid (derived from the cofactor UDPGA) to the substrate, resulting in the generation of the conjugated metabolite (or glucuronide) (Wu et al. 2015; Lu et al. 2017).

The *in vivo* hepatic clearance was predicted and the metabolic stability with different liver microsomes was assessed in this study. According to the values of CL_H_ predicted from the *in vitro* data, compounds can be classified as high-clearance drugs (>70% liver blood flow), low-clearance drugs (<30% liver blood flow) and intermediate-clearance drugs (Li et al. 2003). Toddalolactone was classified as a low-clearance drug in humans, dogs, and rats. The result was also confirmed using a metabolic stability assay. Toddalolactone metabolic stabilities in phase I (CYPs) and II (UGTs) reaction systems were investigated by incubating toddalolactone with different liver microsomes. *t*_1/2_ values were calculated and used to evaluate its metabolite stabilities. Overall, toddalolactone exhibited good metabolic stability in both oxidative metabolism and glucuronidation due to its high *t*_1/2_ value, implying an improved clinical effect and development prospect for toddalolactone by avoiding the rapid metabolism by human metabolic enzymes. In addition, monkeys showed stronger metabolic ability compared with other species. Dogs showed weaker metabolic ability in both oxidative metabolism and glucuronidation. The metabolic characteristics of toddalolactone in rats and mice, as the two most commonly used animal models, were opposite to those in humans. Humans showed weaker metabolic ability compared with rats and mice in the oxidative metabolism of toddalolactone (*p* < 0.05). In contrast, humans showed stronger metabolic ability in toddalolactone glucuronidation compared with rats and mice (*p* < 0.05). In addition, we found that humans and rabbits have similar metabolic characteristics in phase I (CYPs) and II (UGTs) reaction systems according to the results of metabolic stability assessment with different liver microsomes, and thus rabbits may be a good animal model for studying toddalolactone metabolism.

The differences in the contents and activities of CYP enzymes among different species give rise to species-related differences in P450-catalyzed drug oxidation reactions. For example, CYP1A2 accounts for 13% of the total CYP content in the human liver and participates in the metabolism of 4% of drugs on the market. CYP1A2 is expressed at low levels in the livers of monkeys and dogs (Rendic and Di Carlo 1997). Thus, the identification of CYP isoforms involved in the biotransformation of toddalolactone is crucial for assessing species-specific differences in toddalolactone metabolism. Recombinant CYP450 isozymes were used to identify the CYP isoforms involved in the toddalolactone metabolism. These enzymes are expressed in *Saccharomyces cerevisiae* (yeast) with human NADPH-cytochrome P450 reductase and validated as surrogates to their counterparts in human liver microsomes (HLMs). Thus, using recombinant CYP proteins may be a good method to assess the details of toddalolactone metabolism. As shown in Figures 7 and 8, the main metabolite, P4, in human liver microsomes was generated by CYP1A1 and CYP3A5. Since CYP1A1 is poorly expressed in the human liver (Rendic and Di Carlo 1997), CYP3A5 can be considered as the major enzyme to metabolize toddalolactone in the human liver. Humans have a relatively weaker ability to metabolize toddalolactone to produce P4 compared with monkeys. Thus, monkeys show much higher affinity and enzymatic rates for CYP3A substrates compared with humans. For example, cynomolgus monkeys showed fivefold higher CYP3A activity to catalyze midazolam 1′-hydroxylation compared with humans. In addition, monkeys exhibited 19-fold higher CYP3A activity to catalyze erythromycin *N*-demethylation compared with humans (Martignoni et al. 2006). Quinidine, here used for CYP2D6 inhibition, is also an inhibitor of 1A1 (Ching et al. 2001). The use of quinidine did not significantly reduce the P4 formation due to the low level of CYP1A1 in the human liver. Some drugs, such as dasatinib, nicotine, TSU-68, insulin, can induce the expression or activity of CYP1A1 (Kitamura et al. 2008; Wang et al. 2009; Alsaad 2018; Kuzgun et al. 2020). If toddalolactone is co-administered with one of the above drugs, the two drugs may have a synergistic effect to induce CYP1A1, which may lead to adverse effects. When toddalolactone is co-administered with a CYP1A1 substrate such as lenvatinib or phenacetin (Huang et al. 2012; Vavrova et al. 2022), the induction of CYP1A1 by toddalolactone will decrease the concentration of substrate and further reduce the clinical effects. Thus, further research focussed on CYP1A1-mediated DDIs is needed.

Molecular docking and dynamics simulations were used to explore the molecular mechanism underlying the interactions between toddalolactone and CYPs. Toddalolactone bound to the active cavity of CYP1A1 with PHE224 through π–π stacking interactions, and CYP3A5 with GLU374 via a hydrogen bond. PHE224 and GLU374 are key amino acids with the selectivity and region specificity of substrate binding in CYP1A1 and CYP3A5, respectively (Xue et al. 2019; Bart et al. 2020; Santes-Palacios et al. 2020). MD simulations also proved that toddalolactone formed strong and stable interactions with the catalytic cavities of CYP1A1 and CYP3A5 via π-π stacking and hydrogen bond interactions, respectively. Toddalolactone was metabolized by CYP1A1 and CYP3A5 through stable receptor-ligand interactions, and PHE224 in CYP1A1 and GLU374 in CYP3A5 are major players for high catalytic efficiency towards toddalolactone. Western blotting proved that toddalolactone showed no effect on the protein expression of CYP3A5, but significantly induced protein expression of CYP1A1 in a concentration-dependent manner.

This pharmacokinetic study was performed after the intravenous administration of toddalolactone in rats, and toddalolactone was metabolized by rats with a *t*_1/2_ of 1.05 h. Zhou et al. (2021) reported that toddalolactone was eliminated from mouse blood with a *t*_1/2_ of 0.8 h with intravenous administration. According to the metabolic stability results of this study, mice have a more rapidly metabolic rate. Recombinant CYP450 isozymes were used to identify the CYP isoforms involved in the toddalolactone metabolism. These enzymes are expressed in *S. cerevisiae* with human NADPH-cytochrome P450 reductase and validated as surrogates to their counterparts in human liver microsomes (HLMs). Thus, using recombinant CYP proteins may be a good method to assess the details of toddalolactone metabolism. with toddalolactone than rats in phase I (CYPs) reaction system.

## Conclusions

This *in vitro* study demonstrates that the metabolic profiles of toddalolactone exhibited significant differences among species, and monkeys showed the greatest metabolic capacity compared with other species; rabbits displayed metabolic characteristics similar to that of humans. The assessment of metabolism stability indicated good stability of toddalolactone among the various species in CYP-mediated and UGT-mediated reactions. Recombinant human CYP1A1 and CYP3A5 catalyzed the oxidation of toddalolactone, which can be used to predict the metabolic characteristics of toddalolactone among the species due to different levels of expression and activities of CYPs in different species.

## References

[CIT0001] Alsaad A. 2018. Dasatinib induces gene expression of CYP1A1, CYP1B1, and cardiac hypertrophy markers (BNP, beta-MHC) in rat cardiomyocyte H9c2 cells. Toxicol Mech Methods. 28(9):678–684. 29975149 10.1080/15376516.2018.1497746

[CIT0002] Bart AG, Takahashi RH, Wang X, Scott EE. 2020. Human cytochrome P450 1A1 adapts active site for atypical nonplanar substrate. Drug Metab Dispos. 48(2):86–92. 31757797 10.1124/dmd.119.089607 PMC6964148

[CIT0003] Ching MS, Blake CL, Malek NA, Angus PW, Ghabrial H. 2001. Differential inhibition of human CYP1A1 and CYP1A2 by quinidine and quinine. Xenobiotica. 31(11):757–767. 11765139 10.1080/00498250110065603

[CIT0004] Dai ZR, Ning J, Sun GB, Wang P, Zhang F, Ma HY, Zou LW, Hou J, Wu JJ, Ge GB, et al. 2019. Cytochrome P450 3A enzymes are key contributors for hepatic metabolism of bufotalin, a natural constitute in Chinese medicine Chansu. Front Pharmacol. 10:52. 30778299 10.3389/fphar.2019.00052 PMC6369212

[CIT0005] Guo B, Fang Z, Yang L, Xiao L, Xia Y, Gonzalez FJ, Zhu L, Cao Y, Ge G, Yang L, et al. 2015. Tissue and species differences in the glucuronidation of glabridin with UDP-glucuronosyltransferases. Chem Biol Interact. 231:90–97. 25765239 10.1016/j.cbi.2015.03.001 PMC6300978

[CIT0006] He G, Troberg J, Lv X, Xia YL, Zhu LL, Ning J, Ge GB, Finel M, Yang L. 2018. Identification and characterization of human UDP-glucuronosyltransferases responsible for xanthotoxol glucuronidation. Xenobiotica. 48(2):109–116. 28689454 10.1080/00498254.2017.1283719

[CIT0007] He N, Wang P, Wang P, Ma C, Kang W. 2018. Antibacterial mechanism of chelerythrine isolated from root of *Toddalia asiatica* (Linn) Lam. BMC Complement Alternat Med. 18(1):261. 10.1186/s12906-018-2317-3 PMC6158911 30257662

[CIT0008] Huang M, Ho PC. 2009. Identification of metabolites of meisoindigo in rat, pig and human liver microsomes by UFLC-MS/MS. Biochem Pharmacol. 77(8):1418–1428. 19426681 10.1016/j.bcp.2009.01.012

[CIT0009] Huang Q, Deshmukh RS, Ericksen SS, Tu Y, Szklarz GD. 2012. Preferred binding orientations of phenacetin in CYP1A1 and CYP1A2 are associated with isoform-selective metabolism. Drug Metab Dispos. 40(12):2324–2331. 22949628 10.1124/dmd.112.047308 PMC3500547

[CIT0010] Ishii H, Tan S, Wang JP, Chen IS, Ishikawa T. 1991. Studies on the chemical constituents of rutaceous plants. LXVII. The chemical constituents of *Toddalia asiatica* (L.) Lam. (*T. aculeata* Pers). Examination of coumarins using supercritical fluid and Soxhlet extraction. Is toddalolactone a genuine natural coumarin. Yakugaku Zasshi. 111(7):376–385. 1783986 10.1248/yakushi1947.111.7_376

[CIT0011] Jiang H, Meng X, Shi X, Yang J. 2020. Interspecies metabolic diversity of artocarpin *in vitro* mammalian liver microsomes. Biosci Biotechnol Biochem. 84(4):661–669. 31829112 10.1080/09168451.2019.1701405

[CIT0012] Kitamura R, Matsuoka K, Nagayama S, Otagiri M. 2008. Time-dependent induction of rat hepatic CYP1A1 and CYP1A2 expression after single-dose administration of the anti-angiogenic agent TSU-68. Drug Metab Pharmacokinet. 23(6):421–427. 19122336 10.2133/dmpk.23.421

[CIT0013] Korzekwa KR, Krishnamachary N, Shou M, Ogai A, Parise RA, Rettie AE, Gonzalez FJ, Tracy TS. 1998. Evaluation of atypical cytochrome P450 kinetics with two-substrate models: evidence that multiple substrates can simultaneously bind to cytochrome P450 active sites. Biochemistry-US. 37(12):4137–4147. 10.1021/bi9715627 9521735

[CIT0014] Kumagai M, Watanabe A, Yoshida I, Mishima T, Nakamura M, Nishikawa K, Morimoto Y. 2018. Evaluation of aculeatin and toddaculin isolated from *Toddalia asiatica* as anti-inflammatory agents in LPS-stimulated RAW264 macrophages. Biol Pharm Bull. 41(1):132–137. 29311475 10.1248/bpb.b17-00607

[CIT0015] Kuzgun G, Başaran R, Arıoğlu İnan E, Can Eke B. 2020. Effects of insulin treatment on hepatic CYP1A1 and CYP2E1 activities and lipid peroxidation levels in streptozotocin-induced diabetic rats. J Diabetes Metab Disord. 19(2):1157–1164. 33520832 10.1007/s40200-020-00616-y PMC7843681

[CIT0016] Lakshmi V, Kapoor S, Pandey K, Patnaik GK. 2002. Spasmolytic activity of *Toddalia asiatica* var. *floribunda*. Phytother Res. 16(3):281–282. 12164278 10.1002/ptr.844

[CIT0017] Li W, Liu Y, He Y-Q, Zhang J-W, Gao Y, Ge G-B, Liu H-X, Huo H, Liu H-T, Wang L-M, et al. 2008. Characterization of triptolide hydroxylation by cytochrome P450 in human and rat liver microsomes. Xenobiotica. 38(12):1551–1565. 18982531 10.1080/00498250802503359

[CIT0018] Li X, Qiu Z, Jin Q, Chen G, Guo M. 2018. Cell cycle arrest and apoptosis in HT-29 cells induced by dichloromethane fraction from *Toddalia asiatica* (L.) Lam. Front Pharmacol. 9:629. 29950999 10.3389/fphar.2018.00629 PMC6008524

[CIT0019] Li X-Q, Björkman A, Andersson TB, Gustafsson LL, Masimirembwa CM. 2003. Identification of human cytochrome P(450)s that metabolise anti-parasitic drugs and predictions of *in vivo* drug hepatic clearance from *in vitro* data. Eur J Clin Pharmacol. 59(5–6):429–442. 12920490 10.1007/s00228-003-0636-9

[CIT0020] Lu D, Liu H, Ye W, Wang Y, Wu B. 2017. Structure- and isoform-specific glucuronidation of six curcumin analogs. Xenobiotica. 47(4):304–313. 27324181 10.1080/00498254.2016.1193264

[CIT0021] Ma HY, Yang JD, Hou J, Zou LW, Jin Q, Hao DC, Ning J, Ge GB, Yang L. 2017. Comparative metabolism of DDAO benzoate in liver microsomes from various species. Toxicol in Vitro. 44:280–286. 28647665 10.1016/j.tiv.2017.06.020

[CIT0022] Martignoni M, Groothuis GM, de Kanter R. 2006. Species differences between mouse, rat, dog, monkey and human CYP-mediated drug metabolism, inhibition and induction. Expert Opin Drug Metab Toxicol. 2(6):875–894. 17125407 10.1517/17425255.2.6.875

[CIT0023] Matal J, Jancova P, Siller M, Masek V, Anzenbacherova E, Anzenbacher P. 2008. Interspecies comparison of the glucuronidation processes in the man, monkey, pig, dog and rat. Neuro Endocrinol Lett. 29(5):738–743. 18987594

[CIT0024] Mi BL, Sun Q, Qu YQ, Gao XX, Yu ZW, Ge GB, Cai SS, Zhang J, Zheng YC, Zhang ZQ. 2014. Glucuronidation of aurantio-obtusin: identification of human UDP-glucuronosyltransferases and species differences. Xenobiotica. 44(8):716–721. 24618000 10.3109/00498254.2014.895881

[CIT0025] Murugan K, Venus JSE, Panneerselvam C, Bedini S, Conti B, Nicoletti M, Sarkar SK, Hwang J-S, Subramaniam J, Madhiyazhagan P, et al. 2015. Biosynthesis, mosquitocidal and antibacterial properties of *Toddalia asiatica*-synthesized silver nanoparticles: do they impact predation of guppy *Poecilia reticulata* against the filariasis mosquito *Culex quinquefasciatus*? Environ Sci Pollut Res Int. 22(21):17053–17064. 26122577 10.1007/s11356-015-4920-x

[CIT0026] Naritomi Y, Terashita S, Kimura S, Suzuki A, Kagayama A, Sugiyama Y. 2001. Prediction of human hepatic clearance from *in vivo* animal experiments and *in vitro* metabolic studies with liver microsomes from animals and humans. Drug Metab Dispos. 29(10):1316–1324. 11560875

[CIT0027] Ni J, Zhao Y, Su J, Liu Z, Fang S, Li L, Deng J, Fan G. 2020. Toddalolactone protects lipopolysaccharide-induced sepsis and attenuates lipopolysaccharide-induced inflammatory response by modulating HMGB1-NF-kappaB translocation. Front Pharmacol. 11:109. 32153412 10.3389/fphar.2020.00109 PMC7047824

[CIT0028] Oesch F, Fabian E, Landsiedel R. 2018. Xenobiotica-metabolizing enzymes in the skin of rat, mouse, pig, guinea pig, man, and in human skin models. Arch Toxicol. 92(8):2411–2456. 29916051 10.1007/s00204-018-2232-x PMC6063329

[CIT0029] Rendic S, Di Carlo FJ. 1997. Human cytochrome P450 enzymes: a status report summarizing their reactions, substrates, inducers, and inhibitors. Drug Metab Rev. 29(1–2):413–580. 9187528 10.3109/03602539709037591

[CIT0030] Santes-Palacios R, Marroquin-Perez AL, Hernandez-Ojeda SL, Camacho-Carranza R, Govezensky T, Espinosa-Aguirre JJ. 2020. Human CYP1A1 inhibition by flavonoids. Toxicol in Vitro. 62:104681. 31655123 10.1016/j.tiv.2019.104681

[CIT0031] Seibert E, Tracy TS. 2014. Different enzyme kinetic models. Methods Mol Biol. 1113:23–35. 24523107 10.1007/978-1-62703-758-7_3

[CIT0032] Shi X, Zhang G, Ge G, Guo Z, Song Y, Su D, Shan L. 2019. *In vitro* metabolism of auriculasin and its inhibitory effects on human cytochrome P450 and UDP-glucuronosyltransferase enzymes. Chem Res Toxicol. 32(10):2125–2134. 31515991 10.1021/acs.chemrestox.9b00307

[CIT0033] Shi X, Zhang G, Mackie B, Yang S, Wang J, Shan L. 2016. Comparison of the *in vitro* metabolism of psoralidin among different species and characterization of its inhibitory effect against UDP-glucuronosyltransferase (UGT) or cytochrome p450 (CYP450) enzymes. J Chromatogr B Analyt Technol Biomed Life Sci. 1029–1030:145–156. 10.1016/j.jchromb.2016.06.031 27428458

[CIT0034] Shimada T, Mimura M, Inoue K, Nakamura S, Oda H, Ohmori S, Yamazaki H. 1997. Cytochrome P450-dependent drug oxidation activities in liver microsomes of various animal species including rats, guinea pigs, dogs, monkeys, and humans. Arch Toxicol. 71(6):401–408. 9195021 10.1007/s002040050403

[CIT0035] Straniero S, Laskar A, Savva C, Hardfeldt J, Angelin B, Rudling M. 2020. Of mice and men: murine bile acids explain species differences in the regulation of bile acid and cholesterol metabolism. J Lipid Res. 61(4):480–491. 32086245 10.1194/jlr.RA119000307 PMC7112145

[CIT0036] Tang S, Chen A, Zhou X, Zeng L, Liu M, Wang X. 2017. Assessment of the inhibition risk of shikonin on cytochrome P450 via cocktail inhibition assay. Toxicol Lett. 281:74–83. 28941798 10.1016/j.toxlet.2017.09.014

[CIT0037] Tang ZH, Liu YB, Ma SG, Li L, Li Y, Jiang JD, Qu J, Yu SS. 2016. Antiviral spirotriscoumarins A and B: two pairs of oligomeric coumarin enantiomers with a spirodienone-sesquiterpene skeleton from *Toddalia asiatica*. Org Lett. 18(19):5146–5149. 27673343 10.1021/acs.orglett.6b02572

[CIT0038] Vavrova K, Indra R, Pompach P, Heger Z, Hodek P. 2022. The impact of individual human cytochrome P450 enzymes on oxidative metabolism of anticancer drug lenvatinib. Biomed Pharmacother. 145:112391. 34847475 10.1016/j.biopha.2021.112391

[CIT0039] Wang J, Buchman CD, Seetharaman J, Miller DJ, Huber AD, Wu J, Chai SC, Garcia-Maldonado E, Wright WC, Chenge J, et al. 2021. Unraveling the structural basis of selective inhibition of human cytochrome P450 3A5. J Am Chem Soc. 143(44):18467–18480. 34648292 10.1021/jacs.1c07066 PMC8594567

[CIT0040] Wang T, Chen M, Yan YE, Xiao FQ, Pan XL, Wang H. 2009. Growth retardation of fetal rats exposed to nicotine *in utero*: possible involvement of CYP1A1, CYP2E1, and P-glycoprotein. Environ Toxicol. 24(1):33–42. 18442069 10.1002/tox.20391

[CIT0041] Wang YQ, Shang XF, Wang L, Zhang P, Zou LW, Song YQ, Hao DC, Fang SQ, Ge GB, Tang H. 2020. Interspecies variation of clopidogrel hydrolysis in liver microsomes from various mammals. Chem Biol Interact. 315:108871. 31669218 10.1016/j.cbi.2019.108871

[CIT0042] Wei L, Locuson CW, Tracy TS. 2007. Polymorphic variants of CYP2C9: mechanisms involved in reduced catalytic activity. Mol Pharmacol. 72(5):1280–1288. 17686967 10.1124/mol.107.036178

[CIT0043] Wright WC, Chenge J, Wang J, Girvan HM, Yang L, Chai SC, Huber AD, Wu J, Oladimeji PO, Munro AW, et al. 2020. Clobetasol propionate is a heme-mediated selective inhibitor of human cytochrome P450 3A5. J Med Chem. 63(3):1415–1433. 31965799 10.1021/acs.jmedchem.9b02067 PMC7087482

[CIT0044] Wu Z, Liu H, Wu B. 2015. Regioselective glucuronidation of gingerols by human liver microsomes and expressed UDP-glucuronosyltransferase enzymes: reaction kinetics and activity correlation analyses for UGT1A9 and UGT2B7. J Pharm Pharmacol. 67(4):583–596. 25496264 10.1111/jphp.12351

[CIT0045] Xue Y, Li J, Wu Z, Liu G, Tang Y, Li W. 2019. Computational insights into the different catalytic activities of CYP3A4 and CYP3A5 toward schisantherin E. Chem Biol Drug Des. 93(5):854–864. 30637977 10.1111/cbdd.13475

[CIT0046] Yu B, Zhang G, Jin L, Zhang B, Yan D, Yang H, Ye Z, Ma T. 2017. Inhibition of PAI-1 activity by toddalolactone as a mechanism for promoting blood circulation and removing stasis by Chinese herb *Zanthoxylum nitidum* var. *tomentosum*. Front Pharmacol. 8:489. 28785222 10.3389/fphar.2017.00489 PMC5519579

[CIT0047] Zhang G, Hu Y, Pan J. 2014. Interaction between toddalolatone and human serum albumin. J Solution Chem. 43(4):727–745.

[CIT0048] Zhang X, Sun W, Yang Z, Liang Y, Zhou W, Tang L. 2017. Hemostatic chemical constituents from natural medicine *Toddalia asiatica* root bark by LC-ESI Q-TOF MS(E). Chem Cent J. 11(1):55. 29086834 10.1186/s13065-017-0283-3 PMC5472640

[CIT0049] Zhou J, Li Z, Zhang J, Wang H, Yin S, Du J. 2019. 8-Acetonyldihydronitidine inhibits the proliferation of human colorectal cancer cells via activation of p53. Eur J Pharmacol. 854:256–264. 30928633 10.1016/j.ejphar.2019.03.042

[CIT0050] Zhou J, Wang H, Miao C, Yao Y, Ma J. 2021. Development of a rapid UPLC-MS/MS method for the determination of toddalolactone in mouse blood and its application in pharmacokinetics. AChrom. 34(1):18–23.

[CIT0051] Zielinski J, Mevissen M. 2015. Inhibition of *in vitro* metabolism of testosterone in human, dog and horse liver microsomes to investigate species differences. Toxicol in Vitro. 29(3):468–478. 25561246 10.1016/j.tiv.2014.12.018

